# Exosomal microRNA‐26b‐5p down‐regulates ATF2 to enhance radiosensitivity of lung adenocarcinoma cells

**DOI:** 10.1111/jcmm.15402

**Published:** 2020-05-31

**Authors:** Fushi Han, Dongdong Huang, Xinghong Huang, Wei Wang, Shusong Yang, Shuzhen Chen

**Affiliations:** ^1^ Department of Nuclear Medicine Tongji Hospital Tongji University School of Medicine Shanghai China; ^2^ Department of Emergency Medicine Shanghai Pulmonary Hospital Tongji University School of Medicine Shanghai China; ^3^ Department of Radiology Tongji Hospital Tongji University School of Medicine Shanghai China; ^4^ Department of Internal Medicine Tongji Hospital Tongji University School of Medicine Shanghai China; ^5^ Department of Radiotherapy Tongji Hospital Tongji University School of Medicine Shanghai China

**Keywords:** activating transcription factor 2, exosomes, lung adenocarcinoma, lung adenocarcinoma cells, microRNA‐26b‐5p, radiosensitivity

## Abstract

Lung adenocarcinoma (LUAD), as the most common subtype of non‐small cell lung cancer, is responsible for more than 500 000 deaths worldwide annually. In this study, we identify a novel microRNA‐26b‐5p (miR‐26b‐5p) and elucidated its function on LUAD. The survival rate of parent LUAD cells and radiation‐resistant LUAD cells were determined using clonogenic survival assay. We overexpressed or inhibited miR‐26b‐5p in LUAD, and the correlation between activating transcription factor 2 (ATF2) and miR‐26b‐5p was determined using integrated bioinformatics analysis and dual‐luciferase reporter gene assay. Exosomes derived from A549 cell lines were then detected using Western blot assay, followed by co‐transfection with radiation‐resistant A549R cells. LUAD tissues and serum were collected, followed by miR‐26b‐5p relative expression quantification using RT‐qPCR. miR‐26b‐5p was identified as the most differentially expressed miRNA and was down‐regulated in LUAD. Radiation‐resistant cells were more resistant to X‐radiation compared with parent cells. miR‐26b‐5p overexpression and X‐irradiation led to enhanced radiosensitivity of LUAD cells. ATF2 was negatively targeted by miR‐26b‐5p. Exosomal miR‐26b‐5p derived from A549 cells could be transported to irradiation‐resistant LUAD cells and inhibit ATF2 expression to promote DNA damage, apoptosis and radiosensitivity of LUAD cells, which was verified using serum‐based miR‐26b‐5p. Our results show a regulatory network of miR‐26b‐5p on radiosensitivity of LUAD cells, which may serve as a non‐invasive biomarker for LUAD.

## 1 INTRODUCTION

Lung cancer contributed to high incidence and mortality rate and lung adenocarcinoma (LUAD) is the most common histological subtype.^1^, ^2^ Patients with LUAD were found to be subject to greater mortality and the risk of distant metastasis exceeds that of local recurrence at every disease stage, pinpointing the systemic threat of the disease.^3^ Radiotherapy remained to be the primary treatment methods for LUAD either alone or in combination,^4^, ^5^ but radio‐resistance was found in non‐small cell lung cancer leading to decreased efficiency of radiotherapy with corresponding tumour recurrence and metastasis.^6^ In addition, low efficacy of radiotherapy has also been found in patients treated with tyrosine kinase inhibitors for brain metastases from epidermal growth factor receptor‐mutant LUAD.^7^

MicroRNAs (miRNAs) are small non‐coding RNAs that regulate gene expression, and miRNA‐based anticancer therapies are achievable with the goal to improve treatment response and cure rate.^8^ Critical role of miRNAs in cancer pathogenesis and response to therapy have been previously demonstrated in different cancers.^9^, ^10^ A previous study noted that exosomal miR‐26b‐5p was down‐regulated in LUAD and its tumour‐suppressive role was also investigated in bladder cancer by inhibiting cell aggressiveness.^11^, ^12^ miR‐26b‐5p was also found to be involved in proliferation, angiogenesis and apoptosis in hepatocellular carcinoma.^13^ A prior study has demonstrated that exosomes can be derived from LUAD cells^14^ and exosomal miRNAs were found to be a potential biomarker in cancers.^15^ For example, exosomal miR‐451a was identified as a non‐invasive biomarker for early prediction of recurrence and prognosis of non‐small cell lung cancer.^16^ Interestingly, activating transcription factor 2 (ATF2) was predicted to be one of the target genes of miR‐26b‐5p by TargetScan. ATF2 contributed to multiple cellular functions, from development to cellular responses to stresses, particularly in hypoxia and DNA damage response.^17^ ATF2 was involved in suppressing human non‐small cell lung cancer and had effect on melanoma metastasis.^18^, ^19^ In this study, we examined the role of exosomal miR‐26b‐5p derived from LUAD cells in LUAD and its interaction with ATF2. Furthermore, radiation is known to result in DNA double‐strand breaks, which lead to the formation of phosphorylated H2AX (γH2AX) foci, and γH2AX is required for DNA damage signalling and DNA repair.^20^, ^21^, ^22^ Additionally, poly ADP‐ribose polymerase (PARP) enzymes are also implicated in cellular response to DNA damage.^23^, ^24^ Accumulating evidence shed light on Cleaved‐Caspase 3 as a biomarker of tumour cell apoptosis.^25^, ^26^ Therefore, experiments were designed to explore the regulatory mechanism of miR‐26b‐5p/ATF2 in LUAD by using γH2AX, PARP and Cleaved‐Caspase 3 as detection indicators.

## 2 MATERIALS AND METHODS

### 2.1 Ethics statement

All participants agreed with this study and signed written informed consents. The current study was conducted under the International Ethical Guidelines for Biomedical Research Involving Human Subjects with the approval of the Ethics Committee of Shanghai Pulmonary Hospital affiliated to Tongji University School of Medicine (Shanghai, China) and carried out in accordance with the *Declaration of Helsinki*.

### 2.2 Samples

From January 2011 to January 2014, 74 LUAD tissues and adjacent normal tissues (more than 5 cm from the tumours) from patients (18 to 80 years) were collected. All patients were pathologically diagnosed and underwent therapeutic surgery in Shanghai Pulmonary Hospital affiliated to Tongji University School of Medicine. None of them had previous history of treatment with the tumours or presence of excised tumours with negative margins.

Serum samples were collected from 46 clinical patients diagnosed with LUAD and 15 serum samples from healthy individuals were used as controls. Operation was conducted 4‐6 weeks after preoperative radiotherapy, and then, pathological diagnosis was used to evaluate pathological reaction. From 2011 to 2012, 28 patients only received radiotherapy.

### 2.3 Radiation‐resistant cell culture

Three human LUAD cell lines (HCC827, NCI‐H1395 and A549) were purchased from cell bank of Chinese Academy of Sciences (Shanghai, China) while another LUAD cell line SPC‐A1, a normal human pulmonary epithelial cell line (HBE) and HEK293T cells were purchased from BNCC (Kunshan). Radiation‐resistant cell lines were built as previously described.^27^ Specifically, when the cell confluence reached 50%, SPC‐A1, HCC827, NCI‐H1395 and A549 cells were exposed to X‐radiation (2 Gy) and incubated followed by trypsinization at 90% confluence, which was then re‐irradiated when cells reached 50% confluence. Irradiation was repeated 30 times reaching 60 Gy in total. Parent cells were cultured in the same environment without irradiation. In addition, 4‐ or 5‐month intervals were spared between the last two fractional irradiations. Cells exposed to X‐radiation were used in the following experiment after recovery for 2‐3 weeks upon the last radiation.

### 2.4 Radiation clonogenic survival assay

Cells were seeded in six‐well plates in triplicates. Cells were exposed to X‐radiation (2.0, 4.0, 6.0, and 8.0 Gy) separately, followed by culture for 9‐12 days at 37°C until cell colony became visible. Colonies were then stained with crystal violet and counted under a microscope. The survival rate was calculated as follows: (colony number/gold‐plated cell number) irradiation/(colony number/gold‐plated cell number) non‐irradiation.

### 2.5 Immunofluorescence

Cells were exposed to 6.0 Gy X‐radiation and recovered for 48 hours. The cells were grown on the tunnel of μ‐slide VI (Ibidi). Cells were fixed by polyformaldehyde (4%) for 30 minutes, followed by treatment with 0.2% Triton X‐100 for 10 minutes. The antibody to γH2AX (1:100, Abcam; #ab2893) was diluted using 3% bovine serum albumin and incubated with the permeabilized cells. Following wash procedure using phosphate buffer saline (PBS), the secondary antibody Alexa Fluor 488 goat anti‐rabbit antibody to immunoglobulin G (1:2000, Cell Signaling Technology; #4412S) was then added and incubated for 1 hour. Nucleus was stained using 4′,6‐diamidino‐2‐phenylindole (DAPI), and immunofluorescence images were obtained using laser‐scanning confocal microscopy (Leica Microsystems).

### 2.6 Transfection

Cells were seeded in six‐well plates at the density of 6.0 × 10^5^ cells/well. Overexpression or silencing of miR‐26b‐5p, miR‐21‐5p, miR‐206 or miR‐191‐5p in A549 or HCC827 LUAD cell lines was performed by using Lipofectamine 3000. Plasmids (25 pmol) and transfection reagent (10 μL) were added into each well with the final concentration of 10 pmol/mL, followed by incubation with 5% CO_2_ at 37°C. pcDNA3.1‐ATF2 (100 nmol/L) was transfected into A549 and HCC827 LUAD cell lines as negative controls (NCs) (GenePharma) to investigate the effect of ATF2 on radiosensitivity in LUAD cells. Cells were then used for subsequent experiment after incubation for 48 hours.

### 2.7 Isolation and characterization of exosomes

Exosomes were depleted using foetal bovine serum (FBS) via ultracentrifugation (1 × 10^6^ *g*) for 16 hours at 4°C (Beckman Coulter Avanti J‐30I). Preparation method of FBS without exosomes: The serum was ultracentrifuged at 1 × 10^6^ before centrifugation, and the clarified part of the serum was collected. If the purity was not enough, the serum collected after centrifugation was subjected to secondary ultracentrifugation. After successful preparation, FBS without exosomes was stored at −80°C for later use. Following incubation for 48‐72 hours, medium was then collected and exosomes were isolated using ultracentrifugation. The mixture of an equal volume of plasma (1 mL) and filtered PBS was used to reduce the viscosity of the solution before centrifugation. Medium was centrifuged (300 *g* for 10 minutes; 2000 *g* for 15 minutes; 12 000 *g* for 30 minutes) to discard floating cells and cell debris, followed by filtering using 0.22‐μm filter. Supernatants were ultracentrifuged for 2 hours at 4°C (1 × 10^6^ *g*), and ultracentrifugation was repeated, followed by resuspension in PBS and store at −80°C.

The above exosome precipitation was incubated in 2% glutaraldehyde overnight (4°C), followed by fixation using 1% OsO_4_, dehydration in ethanol and embedding using epoxy resin. Embedded exosome precipitation was then sectioned, followed by addition of saturated sodium periodate and 0.1 N hydrochloric acid. The morphology and ultrastructure of exosomes were analysed by transmission electron microscopy (JEM‐1010; JEOL) 10 minutes later. The particle size of exosomes was analysed by NanoSight nanoparticle tracking analysis (Malvern Panalytical company).

### 2.8 Shuttling assays for exosomes and miR‐26b‐5p

Lipofectamine 2000 reagent (Invitrogen) was used to transfect miR‐26b‐5p labelled by 50 nmol/L Cy3 into A549 cells. Exosomes were retrieved using differential centrifugation after 24 hours incubation, and then, 200 pg exosomes were added with 1 mL Diluent C solution. The mixture of 10 mmol/L 3,3′‐dioctadecyloxacarbocyanine perchlorate (DIO) was added in an Eppendorf tube supplemented with l mL Diluent C, followed by centrifugation for 2 hours (100 000 *g*) at 4°C. Precipitated exosomes were resuspended in Dulbecco's modified Eagle medium containing 10% FBS and penicillin/streptomycin and transferred to recipient A549 cells, and then seeded at the density of 1 × 10^5^ per well in 35 mm dish. Cells were stained with DAPI. The fluorescence images of Cy3‐labelled miR‐26b‐5p and DIO‐labelled exosomes in the recipient cells were observed by a confocal microscopy (Olympus FV1000).

### 2.9 Western blot assay

Total protein was isolated and separated by sodium dodecyl sulphate‐polyacrylamide gel electrophoresis and then transferred onto polyvinylidene fluoride membranes. The membrane was blocked with 5% skim milk for 1 hour and incubated with the diluted antibodies (Table 1). The secondary antibody labelled by horseradish peroxidase was added and then incubated for 1 hour. Enhanced chemiluminescence (BB‐3501, Amersham) was used to develop signals under Gel imager. Photographs were taken using Bio‐Rad image analysis system (BIO‐RAD Laboratories) and then analysed by Quantity One v4.6.2. The relative protein expression was regarded as the density between the protein to be tested and β‐actin.

**Table 1:** Antibodies used for Western blot assay

Antibody	Source	Product information	Used for
Anti‐Cleaved PARP	Rabbit monoclonal	ab32064, Abcam, UK	WB (1:2000)
Anti‐Cleaved Caspase 3	Rabbit polyclone	ab2302, Abcam, UK	WB (1:1000)
Anti‐ATF2	Rabbit polyclone	ab47476, Abcam, UK	WB (1:1000)
Anti‐γH2AX	Mouse monoclone	ab26350, Abcam, UK	WB (1:1000)
Anti‐CD63	Rabbit polyclone	ab216130, Abcam, UK	WB (1:1000)
Anti‐TSG101	Rabbit polyclone	14497, Proteintech, USA	WB (1:1000)
Anti‐HSP70	Rabbit polyclone	4873, Cell Signaling Technology, USA	WB (1:1000)
Anti‐CD9	Mouse monoclone	ab2215, Abcam, UK	WB (1:1000)
Anti‐calnexin	Rabbit polyclone	ab22595, Abcam, UK	WB (1:1000)
Anti‐β‐actin	Mouse monoclone	A5316, Sigma, USA	WB (1:10 000)

Abbreviation: ATF2, activating transcription factor 2; CD9, cluster of differentiation 9; HSP70, heat shock protein 70; PARP, poly ADP‐ribose polymerase; γH2AX, phosphorylated H2AX; TSG101, tumour susceptibility gene 101; WB, Western blot analysis.

### 2.10 Reverse transcription‐quantitative polymerase chain reaction

Total RNA was extracted using TRIzol (16096020, Thermo Fisher Scientific) and reversely transcribed into cDNA by RT‐qPCR kit (ABI). miRNA was reversely transcribed and detected using miScript II RT kit (218161, QIAGEN) and miScript SYBR GreenPCR kit (218075, QIAGEN). Primers are listed in Table 2 with U6 as internal control for miR‐26b‐5p, and β‐actin for other genes. The fold changes were calculated by means of relative quantification (2^−ΔΔCt^ method).

**Table 2:** Primer sequences for RT‐qPCR

Target	Forward (5′‐3′)	Reverse (5′‐3′)
miR‐26b‐5p	TATCTAGACATCTGCTACCTCCTCCC	ATGCGGCCGCGATTCAACAAGGACAA
ATF2	TACAAGTGGTCGTCGG	CGGTTACAGGGCAATC
U6	CTCGCTTCGGCAGCACA	AACGCTTCACGAATTTGCGT
β‐actin	GGGAAATCGTGCGTGACATTAAGG	CAGGAAGGAAGGCTGGAAGAGTG

Abbreviations: ATF2, activating transcription factor 2; miR‐26b‐5p, microRNA‐26b‐5p; RT‐qPCR, reverse transcription quantitative polymerase chain reaction; U6, small nuclear RNA.

### 2.11 Dual‐luciferase reporter gene assay

The 3′ untranslated region (3′UTR) of ATF2 was artificially synthesized. The binding sites between miR‐26b‐5p and 3′UTR of ATF2 was predicted using TargetScan. Site‐directed mutation (mut) was obtained based on the wide type (wt) of 3'UTR of ATF2 using QuikChange Site‐Directed Mutagenesis Kit (Stratagene) with ATF2 3′UTR (UUACUUGAA) and mut (AAUGAACUU) sequences. The above wt and mut inserted were cloned into pNL1.1 (Promega). pRL‐TK of Renilla luciferase was used as internal reference to adjust cell number and transfection efficiency. HEK293T cells were co‐treated with miR‐26b‐5p mimic, mimic‐NC (4464084; ABI) and wt and mut plasmids. The dual‐luciferase activity was measured.

### 2.12 Tumour xenografts in nude mice

BALB/c nude mice (aged 5 weeks) were injected with miR‐26b‐5p overexpressed s, followed by X‐radiation using single‐dose12 Gy when tumour diameter reached 5 mm. Each group consisted of five mice. Calliper was used to measure tumour volume (*V*) 2 days after X‐radiation. Tumours were extracted, measured and photographed. *V* = (LW^2^)/*2* (L = length; W = width).

### 2.13 Statistical analysis

All data were processed and analysed using SPSS 21.0 statistical software (IBM Corp., Armonk). Measurement data were expressed as mean ± standard deviation. Paired/unpaired *t* test was used to analyse differences between normally distributed values of two experimental groups. Differences among normally distributed values of three or more experimental groups were analysed by one‐way analysis of variance (ANOVA), followed by a Tukey's post hoc test. Comparisons between time‐based measurements within each group were performed using ANOVA of repeated measurements, followed by Bonferroni's post‐test. Pearson's correlation analysis was adopted to analyse the correlation between two indicators. The criterion for statistical significance was set at *P* < .05.

## 3 RESULTS

### 3.1 miR‐26b‐5p was repressed in the radiation‐resistant LUAD cells

**Figure 1:**
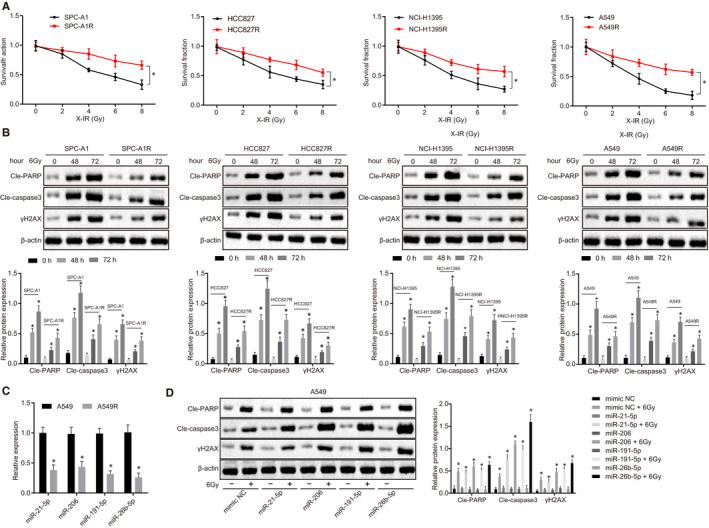
miR‐26b‐5p is down‐regulated in radiation‐resistant LUAD cells. A, Survival rate of cells after radiation using clonogenic survival assay. B, Cleaved‐PARP, Cleaved‐Caspase 3 and γH2AX expression in parental cells and irradiation‐resistant cells normalized to β‐actin determined by Western blot analysis. C, miR‐21‐5p miR‐206, miR‐191‐5p and miR‐26b‐5p expression in A549 and A549R cells determined by RT‐qPCR. D, Effect of miR‐21‐5p miR‐206, miR‐191‐5p and miR‐26‐5p overexpression on Cleaved‐PARP, Cleaved‐Caspase 3 and γH2AX expression in A549 cells normalized to β‐actin compared to mimic‐NC using Western blot analysis. *P < .05; Measurement data were expressed as mean ± standard deviation. Independent sample *t* test was used to analyse differences between two groups. ANOVA of repeated measurements was used in panel A, followed by Bonferroni's post‐test. Experiments were repeated in triplicates

In order to produce radiation‐resistant LUAD cells, we treated LUAD cell lines SPC‐A1, HCC827, NCI‐H1395 and A549 with X‐radiation. As shown in Figure 1A, radiation clonogenic survival assay revealed that radiation‐resistant subgroup (SPC‐A1R, HCC827R, NCI‐H1395R and A549R) exhibited significantly enhanced resistance to X‐radiation in comparison to their parent cells (SPC‐A1, HCC827, NCI‐H1395 and A549).

Western blot assay (Figure 1B) was performed to determine expression of Cleaved‐PARP, Cleaved‐Caspase 3 and γH2AX in parent cells and irradiation‐resistant cells following irradiation. The data demonstrated that Cleaved‐PARP, Cleaved‐Caspase 3 and γH2AX expression increased over time during the irradiation treatment. In addition, significantly lower expression of Cleaved‐PARP, Cleaved‐Caspase 3 and γH2AX was observed in irradiation‐resistant cells compared to their parent cells. Thus, irradiation‐resistant cells exhibit reduced Caspase‐3 and RARP protease activity in the DNA damage signalling in vitro.

To better elucidate the function of miRNAs in radiation sensitivity, miR‐21‐5p, miR‐206, miR‐191‐5p and miR‐26‐5p were selected as potential miRNAs that might affect the progression of non‐small cell lung cancer based on a previous study.^11^ Expression of these miRNAs was determined by RT‐qPCR in A549 and radiation‐resistant A549 (A549R) cells (Figure 1C). miR‐26b‐5p was identified as the most differentially expressed miRNA in A549R cells. The function of miRNA in cell apoptosis was further tested by transfecting miRNAs into A549 cells, followed by exposure to 6.0 Gy X‐radiation. In Figure 1D, the results showed that overexpression of miRNAs led to enhanced Caspase‐3 and RARP protease activity in response to DNA damage and overexpression of miR‐26b‐5p contributed to the greatest up‐regulation of Cleaved‐PARP, Cleaved‐Caspase 3 and γH2AX, suggesting overexpression of miR‐26b‐5p can induce cell apoptosis via these genes, and therefore, miR‐26b‐5p was used for the subsequent experiment.

### 3.2 miR‐26b‐5p overexpression restored radiosensitivity of A549 cells

**Figure 2:**
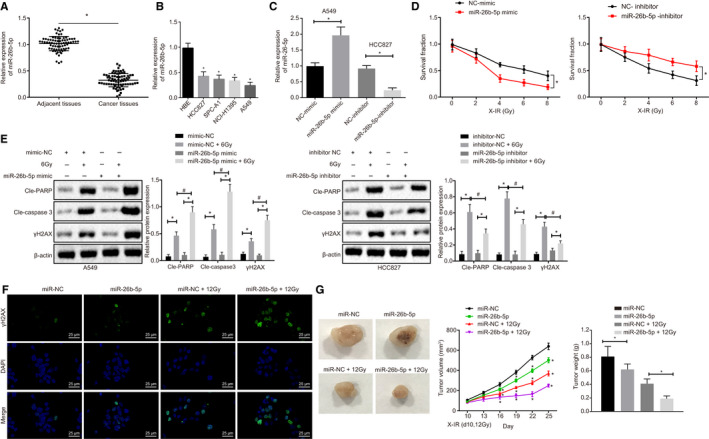
miR‐26b‐5p overexpression enhances radiosensitivity of A549 cells. A, miR‐26b‐5p expression in LUAD tissues and adjacent tissues using RT‐qPCR. B, miR‐26b‐5p expression in SPC‐A1, HCC827, NCI‐H1395 and A549 LUAD cell lines determined by RT‐qPCR. C, miR‐26b‐5p expression in response to miR‐26b‐5p overexpression in A549 cells and miR‐26b‐5p expression in response to miR‐26b‐5p knockdown in HCC827 cells determined by RT‐qPCR. D, Cell proliferation detected by radiation clonogenic survival assay. E, Cleaved‐PARP, Cleaved‐Caspase 3 and γH2AX expression in A549 and HCC827 cell lines normalized to β‐actin using Western blot assay. F, Immunofluorescence assay in γH2AX expression, following miR‐26b‐5p overexpression, bar = 25 µm. G, Overexpression of miR‐26b‐5p in tumour xenografts in nude mice compared with miR‐NC, miR‐NC + 12Gy, miR‐26b‐5p + 12Gy. *^&#^ *P* < .05; Measurement data were expressed as mean ± standard deviation. Paired/unpaired *t* test was used to analyse differences between two groups, and differences among multiple groups were analysed by one‐way ANOVA, followed by Tukey's post hoc tests. ANOVA of repeated measurements was used in panel D and G, followed by Bonferroni's post‐test. Cell experiments were repeated in triplets, n = 46 (patients), n = 5 (mice)

Until now, the modulatory roles of miR‐26b‐5p on LUAD cells to radiosensitivity are not clear. To address this, we measured miR‐26b‐5p expression in LUAD tissues and cells. Down‐regulation of miR‐26b‐5p was found both in LUAD tissues and LUAD cell lines compared to cancer tissues and HBE, respectively (Figure 2A,B). Next, we overexpressed miR‐26b‐5p in A549 cells and performed miR‐26b‐5p knockdown in HCC827 cells to further investigate the relationship between radiosensitivity and miR‐26b‐5p (Figure 2C‐E). The results indicated that miR‐26b‐5p overexpression restored radiosensitivity of A549 cells, and knockdown of miR‐26b‐5p resulted in radioresistance. In addition, in A549 cells, higher PARP, Caspase‐3 and γH2AX expression were observed in response to miR‐26b‐5p overexpression following X‐radiation treatment while in HCC827 cell lines, an opposite trend was shown in response to miR‐26b‐5p inhibition.

Immunofluorescence results are shown in Figure 2F, and we identified elevated γH2AX expression in response to miR‐26b‐5p overexpression following 6.0 Gy X‐radiation in A549 cells, suggesting miR‐26b‐5p promoted radiosensitivity of LUAD cells via DNA damage‐induced cell apoptosis. Our in vivo experiment (Figure 2G) also revealed that miR‐26b‐5p inhibited tumour growth, and particularly, more significantly inhibitory effect on tumour growth was observed in response to overexpression of miR‐26b‐5p following X‐radiation.

### 3.3 ATF2 was targeted by miR‐26b‐5p

**Figure 3:**
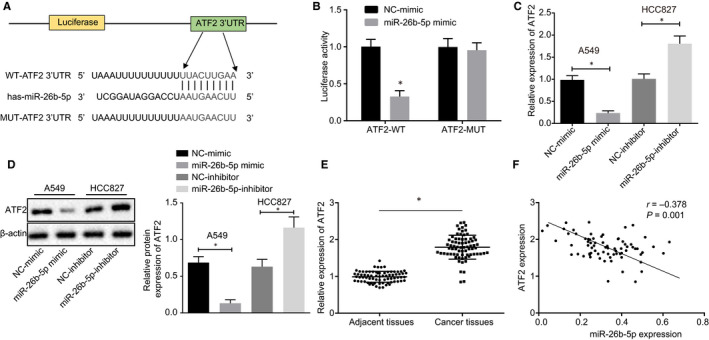
ATF2 is negatively targeted by miR‐26b‐5p. A, The binding sites between ATF2 and miR‐26b‐5p were predicted using TargetScan. B, Specific binding sites between ATF2 and miR‐26b‐5p determined by dual‐luciferase reporter gene assay. C, ATF2 expression in HCC827 and A549 cell lines in response to miR‐26b‐5p mimic and miR‐26b‐5p inhibitor using RT‐qPCR. D, ATF2 protein expression in HCC827 and A549 cell lines normalized to β‐actin in response to miR‐26b‐5p mimic and miR‐26b‐5p inhibitor using Western blot analysis. E, ATF2 expression in LUAD tissues and adjacent tissues determined by RT‐qPCR. F, Correlation analysis between miR‐26b‐5p and ATF2. *^&#^ *P* < .05; Measurement data were expressed as mean ± standard deviation. Paired/unpaired *t* test was used to analyse differences between two groups, and Pearson's correlation analysis was adopted in panel F. Cell experiments were repeated in triplets, n = 46 (patients)

ATF2 was predicted to be one of the target genes of miR‐26b‐5p by TargetScan (http://www.targetscan.org) and mutant binding sites between ATF2 and miR‐26b‐5p were found (Figure 3A). Results of dual‐luciferase reporter gene assay are illustrated in Figure 3B, which showed that luciferase activity significantly decreased in HEK293T cells treated with miR‐26b‐5p mimic and ATF2‐wt (*P* < .05). Luciferase activity experienced no significant change after transfection with ATF2‐mut, suggesting ATF2 can be targeted by miR‐26b‐5p. As shown in Figure 3C,D, we introduced miR‐26b‐5p mimic or miR‐26b‐5p inhibitor to HCC827 and A549 cells, respectively, and the results revealed that ATF2 expression were both decreased in response to miR‐26b‐5p mimic compared to mimic‐NC. On the other hand, the trend was opposite in response to miR‐26b‐5p inhibitor (*P* < .05), which further confirmed that ATF2 specifically served as a target gene for miR‐26b‐5p. ATF2 expression in LUAD tissues and adjacent normal tissues were measured in Figure 3E,F, which suggested that ATF2 expression was elevated in LUAD and was negatively targeted by miR‐26b‐5p. Taken together, ATF2 was negatively targeted by miR‐26b‐5p and miR‐26b‐5p overexpression contributed to repressed ATF2 expression in LUAD cells.

### 3.4 Overexpression of miR‐26b‐5p induced cell apoptosis and enhanced radiosensitivity by inhibiting ATF2

**Figure 4:**
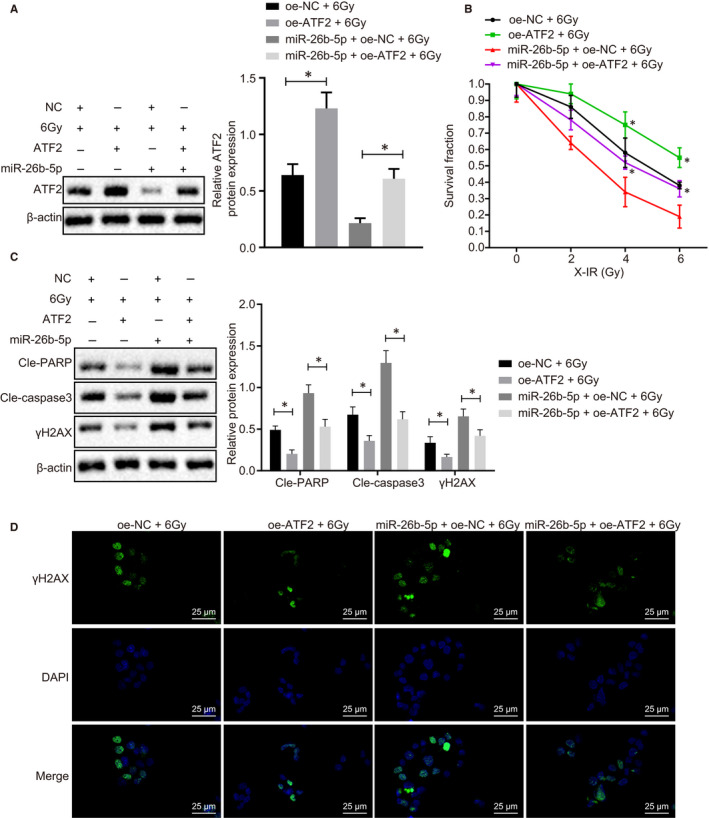
miR‐26b‐5p overexpression induces cell apoptosis and restores radiosensitivity by inhibiting ATF2. A, ATF2 expression in A549 cells normalized to β‐actin in response to oe‐miR‐26b‐5p or oe‐ATF2 determined by Western blot analysis. B, Cell proliferation determined using radiation clonogenic survival assay. C, Cleaved‐PARP, Cleaved‐Caspase 3 and γH2AX expression normalized to β‐actin determined using Western blot analysis. D, Immunofluorescence determination on γH2AX expression following ATF2 overexpression compared with NC, miR‐26b‐5p and miR‐26b‐5p + ATF2 after exposure to 6Gy radiation, bar = 25 µm. *^&#^ *P* < .05; Measurement data were expressed as mean ± standard deviation. Independent sample *t* test was used to analyse differences between two groups, and ANOVA of repeated measurements was used in panel B, followed by Bonferroni's post‐test. Experiments were conducted three times independently

To further elucidate the miR‐26b‐5p and ATF2 function in radiosensitivity of LUAD cells, we overexpressed miR‐26b‐5p or ATF2 in A549 cells and found that ATF2 protein expression elevated in response to oe‐ATF2 compared to oe‐NC. In addition, ATF2 overexpression reversed the effect of miR‐26b‐5p on ATF2 in the Western blot assay (Figure 4A). ATF2‐overexpressed A549 cells exhibited radioresistance and contributed to reduced PARP, Caspase‐3 activity and γH2AX expression following X‐radiation treatment (Figure 4B,C). γH2AX expression in A549 cells was observed as detected by immunofluorescence in Figure 4D, and oe‐ATF2 reduced γH2AX expression after exposure to 6.0 Gy X‐radiation. Meanwhile, the rescue experiment reversed the effect of miR‐26b‐5p on enhanced radiosensitivity of A549 cells. Collectively, miR‐26b‐5p overexpression induced cell apoptosis and restored radiosensitivity by targeting ATF2 in DNA damage.

### 3.5 Exosomal miR‐26b‐5p was transferred to irradiation‐resistant cells

**Figure 5:**
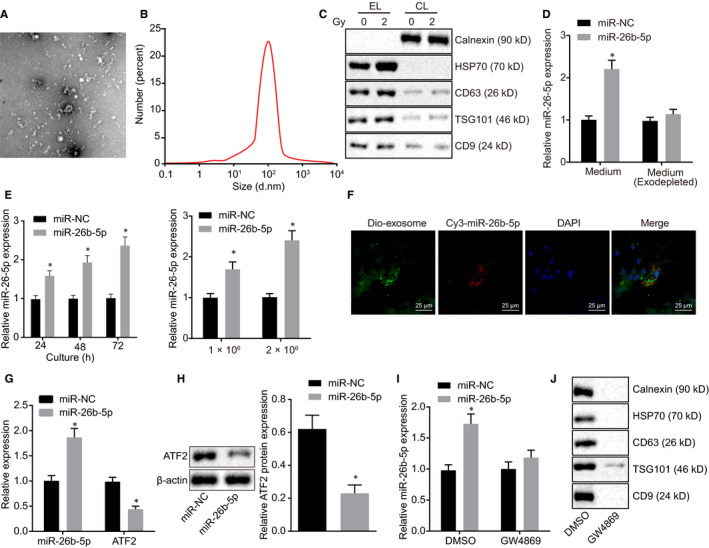
Exosomal miR‐26b‐5p can be transferred in irradiation‐resistant cells. A, Isolated exosomes from A549 observed by transmission electron microscopy. B, Particle size and concentration of exosomes observed using NanoSight N300. C, Western blot determination of protein markers of exosomes in EL and CL. D, RT‐qPCR quantification of miR‐26b‐5p expression in A549 cells in the absence of exosomes. E, miR‐26b‐5p expression at different culture time determined by RT‐qPCR. F, Cellular localization of DIO‐labelled exosomes and Cy3‐labelled miR‐26b‐5p using confocal microscope in A549R cells. G, miR‐26b‐5p and ATF2 expression in A549R cells using RT‐qPCR. H, ATF2 protein expression in A549R cells normalized to β‐actin using Western blot assay. I, miR‐26b‐5p expression in the medium treated with GW4869 or DMSO using RT‐qPCR. J, The exosomal markers detected in A549 cells treated with GW4869 or DMSO using Western blot analysis. *^&#^ *P* < .05; Measurement data were expressed as mean ± standard deviation. Independent sample *t* test was used to analyse differences between two groups, and experiments were conducted three times independently. EL: exosome lysates; CL: cell lysates

To explore whether miR‐26b‐5p can be transferred in LUAD cells via exosomes, we isolated exosomes from A549 cells and transfected them with miR‐26b‐5p mimic. The isolated exosomes were round or oval in shape with 30‐100 nm in diameter with enveloped structure under transmission electron microscopy (Figure 5A). NanoSight N300 was applied to measure particle size and distribution, and then, we identified 30‐150 nm in diameter of exosomes (Figure 5B). Western blot assay (Figure 5C) was used to measure exosome markers (CD63, TSG101 and Alix) and calnexin expression in exosome lysates (EL) and cell lysates (CL). The results identified the existence of CD63, CD9, TSG101 and HSP70 but no signal of calnexin in EL derived from A549 cells post X‐radiation treatment. However, no exosome markers were detected in CL.

Next, we detected miR‐26b‐5p expression in supernatant in the absence of exosomes and observed lower expression of miR‐26b‐5p using RT‐qPCR (Figure 5D). Meanwhile, as shown in Figure 5E, miR‐26b‐5p expression increased over the duration of culture and corresponded to larger number of cells, indicating miR‐26b‐5p was encapsulated in exosomes. Exosomes isolated from Cy3‐labelled A549 cells were labelled with DIO and then cultured with A549R cells for 48 hours. Both red and green fluorescence were observed in the cytoplasm of A549R cells as detected by confocal microscope, indicating A549R cells can absorb exosomes‐encapsulated miR‐26b‐5p (Figure 5F). Expression of miR‐26b‐5p and ATF2 in A549R cells treated with exosomes was then measured using Western blot assay and RT‐qPCR (Figure 5G,H). The results identified higher expression of miR‐26b‐5p, but lower expression of ATF2. In addition, we employed GW4869 to prevent secretion of exosomes and found that GW4869 can inhibit the secretion of exosomes and correlated with reduced miR‐26b‐5p expression (Figure 5I,J). Taken together, these findings demonstrated that miR‐26b‐5p was encapsulated in exosomes and exosomal miR‐26b‐5p down‐regulated the expression of TRAF3 in recipient cells.

### 3.6 Exosomal miR‐26b‐5p transferred to irradiation‐resistant cells induced cell apoptosis and restored radiosensitivity

**Figure 6:**
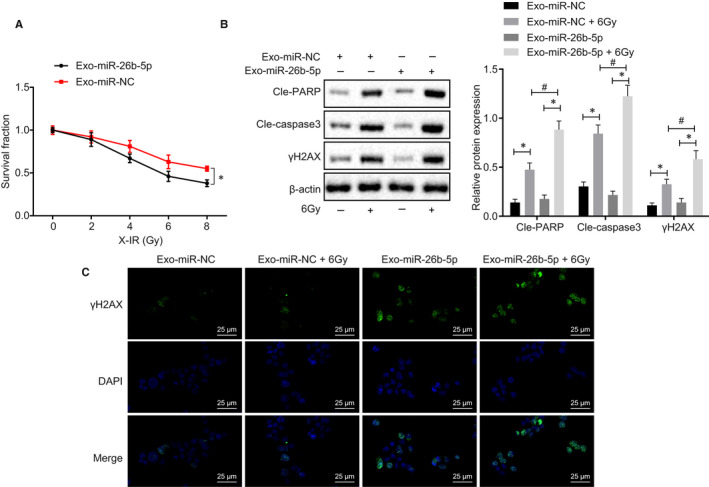
Exosomal miR‐26b‐5p transferred to A549R induces cell apoptosis and restores radiosensitivity. A, Cell proliferation of exo‐miR‐NC and exo‐miR‐26b‐5p determined by radiation clonogenic survival assay. B, Cleaved‐PARP, Cleaved‐Caspase 3 and γH2AX expression normalized to β‐actin in exo‐miR‐NC, 6Gy and exo‐miR‐26b‐5p determined using Western blot assay. C, γH2AX expression in exo‐miR‐NC, exo‐miR‐NC + 6Gy, exo‐miR‐26b‐5p, exo‐miR‐26b‐5p + 6Gy determined by immunofluorescence, bar = 25 µm. *^&#^ *P* < .05; Measurement data were expressed as mean ± standard deviation. Independent sample *t* test was used to analyse differences between two groups, and ANOVA of repeated measurements was used in panel A, followed by Bonferroni's post‐test. Experiments were conducted 3 times independently

We detected radiosensitivity of exosomes‐treated A549R cells and found that A549R cells were more sensitive to irradiation (Figure 6A). As shown in Figure 6B, exosomes‐encapsulated miR‐26b‐5p stimulated PARP, Caspase‐3 activity and γH2AX expression. γH2AX expression was measured in A549R cells treated with exosomal miR‐26b‐5p, and the results showed that γH2AX was induced in response to exosomes‐transported miR‐26b‐5p by immunofluorescence (Figure 6C). Taken together, exosomes‐transported miR‐26b‐5p transferred induced cell apoptosis and restored radiosensitivity in irradiation‐resistant cells.

### 3.7 Exosomal miR‐26b‐5p from serum served as a non‐invasive biomarker for LUAD

**Figure 7:**
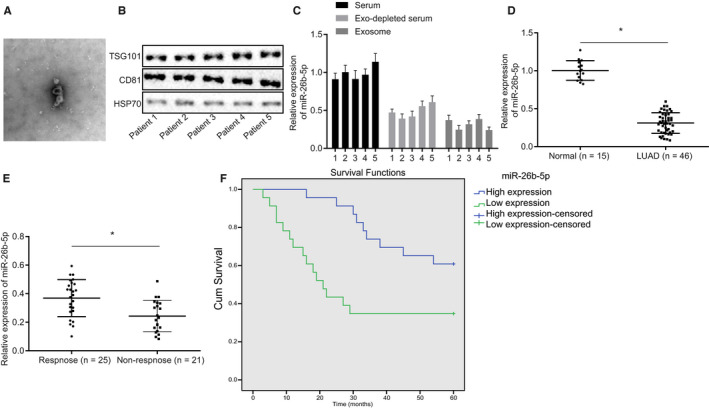
Serum miR‐26b‐5p can serve as a non‐invasive biomarker for LUAD. A, Exosomes isolated from serum of five LUAD patients observed under microscope B, Exosomal markers determined using Western blot analysis. C, miR‐26b‐5p expression in serum and exosomes using RT‐qPCR. D, RT‐qPCR was adopted to measure miR‐26b‐5p expression in LUAD patients (46) and healthy control (15). E, Comparison of miR‐26b‐5p expression between non‐responsive (21) and responsive (25) groups of patient serum samples. F, Survival curve of 46 patients with LUAD with different level of miR‐26b‐5p using Kaplan Meier survival analysis. Measurement data were expressed as mean ± standard deviation. Independent sample *t* test was used to analyse differences between two groups

We hypothesized that exosomal miR‐26b‐5p can be transported to blood. For verification purpose, we isolated serum from five patients with LUAD, followed by purification of exosomes and then Western blot assay was used to test the existence of exosomes (Figure 7A,B). As shown in Figure 7C, miR‐26b‐5p expression was detected in exosomes isolated from LUAD serum. Next, we studied the correlation between serum miR‐26b‐5p expression and clinicopathological parameters and identified that reduced miR‐26b‐5p was found in patients with LUAD compared to healthy control (Figure 7D). Significant reduction of miR‐26b‐5p expression was observed in patients with LUAD without radiotherapy (Figure 7E). In addition, strikingly reduced survival period of patients with LUAD was correlated with low expression of miR‐26b‐5p in serum compared with patients with high expression of miR‐26b‐5p (Figure 7F). Taken together, serum miR‐26b‐5p can be a potential non‐invasive biomarker for radiotherapy response and prognosis of LUAD.

## 4 DISCUSSION

In this study, we found miR‐26b‐5p was down‐regulated and its overexpression was correlated with enhanced radiosensitivity of A549 cells and LUAD cell apoptosis in LUAD tissues. ATF2 overexpression inhibited radiosensitivity, which could be negatively targeted by miR‐26b‐5p in DNA damage. Furthermore, exosomal miR‐26b‐5p transferred to irradiation‐resistant cells induced cell apoptosis and enhanced radiosensitivity by regulating ATF2. Exosomal miR‐26b‐5p from patient serum served as a non‐invasive biomarker for LUAD. These findings indicated that regulatory mechanism of miR‐26b‐5p/ATF2 was a potential therapeutic target in LUAD.

The first finding of this study was a lower level of miR‐26b‐5p found in LUAD tissues than that in adjacent normal tissues. miRNAs were known to be associated with many processes of cellular differentiation and homeostasis, which consequently exert influence on various pathologies, including cancer.^28^ Previous studies have noted the repressive role of miRNAs in prostate cancer and hepatocellular carcinoma.^29^, ^30^ Similar to our study, miR‐26b‐5p was down‐regulated in non‐small cell lung cancer cell lines than that in normal epithelial cells, and its up‐regulation was also found to inhibit proliferation and induce apoptosis of lung cancer cells.^31^ Also, overexpression of miR‐26b‐5p elevated expression of apoptosis‐related proteins caspase 3 and PARP,^32^ to support our findings that miR‐26b‐5p elevation promotes LUAD cell apoptosis. CDP138 was unravelled to enhance radiosensitivity in lung cancer cells by increasing γ‐H2AX foci in CDP138‐depleted lung cancer cells after exposure to radiation,^33^ and γ‐H2AX has been regarded as a useful marker of cellular radiosensitivity after single and fractionated irradiation in vivo.^34^ Likewise, increased expression of γ‐H2AX was induced by miR‐26b‐5p in A549 cells in our study, suggesting that miR‐26b‐5p can enhance radiosensitivity of LUAD cells.

To better understand the specific mechanism underlying how miR‐26b‐5p enhanced radiosensitivity of LUAD cells, we used TargetScan (http://www.targetscan.org) to predict the target gene of miR‐26b‐5p. ATF2 was up‐regulated in LUAD cells, and its overexpression inhibited radiosensitivity and apoptosis. Our interaction assay showed that ATF2 can be targeted by miR‐26b‐5p in the process of DNA damage. Concordantly, the targeting relation between ATF2 and miR‐26b has been validated in γ‐irradiated lung cancer cells.^35^ The crucial functions of activated ATF2 in the therapeutic resistance of melanoma have been previously reported, and Lau E *et al* have reported that ATF2 plays a critical role in melanoma resistance to therapy.^36^ A more recent study also revealed that elevated AFT2 expression in non‐small cell lung cancer cells.^37^ In addition to its transcriptional role, AFT2 was found to be involved in DNA damage by inducing apoptosis.^38^, ^39^

Exosomes contain DNA, RNA, proteins and lipids as small extracellular vesicles. They serve as strong signalling molecules between cancer cells and the surrounding cells. Exosomes transported from either tumour or stromal cells have been shown to be essential in all phases of cancer progression and exert pivotal importance in therapy resistance.^40^ For example, exosomal miR‐1290 and miR‐375 are regarded as promising prognostic biomarkers for patients with castration‐resistant prostate cancer.^41^ A more recent study identified exosomal miR‐451a as a novel biomarker for the early prediction of recurrence and prognosis in patients with non‐small cell lung cancer after curative resection.^16^ We found evidence that exosomal miR‐26b‐5p could enhance radiosensitivity of LUAD cells. Moreover, exosomal miR‐26b‐5p from serum served as a non‐invasive biomarker for LUAD and exosomal miRNAs from serum have previously served as a clinical biomarker in human oesophageal squamous cell carcinoma.^42^

## 5 CONCLUSION

The present study provides new insights into the mechanism of miR‐26b‐5p in the radiosensitivity of LUAD cells. Particularly, miR‐26b‐5p induced LUAD cells apoptosis and enhanced radiosensitivity of LUAD cells by down‐regulating ATF2. This mechanism can serve as a therapeutic and prognosis target for LUAD in the future.

## CONFLICT OF INTEREST

The authors declare that they have no conflict of interest.

## AUTHOR CONTRIBUTION

Fushi Han, and Dongdong Huang wrote the paper and conceived and designed the experiments; Xinghong Huang and Wei Wang analysed the data; Shuzhen Chen and Shusong Yang collected and provided the figures of the study. All the authors reviewed and approved the final version of the paper.

## Data Availability

The data used to support the findings of this study are available from the corresponding author upon request.
